# Hiding scattering layers for noninvasive imaging of hidden objects

**DOI:** 10.1038/srep08375

**Published:** 2015-02-11

**Authors:** Kedi Wu, Qiluan Cheng, Yile Shi, Hui Wang, Guo Ping Wang

**Affiliations:** 1 College of Electronic Science and Technology, Shenzhen University, Shenzhen 518060, China; 2 School of Physics and Technology, Wuhan University, Wuhan 430072, China; 3 Institute of Information Optics, Zhejiang Normal University, Jinhua 321000, Zhejiang, P. R. China

## Abstract

The ability to noninvasive image through turbid media has long been a major scientific and technological goal in many disciplines. A breakthrough has been made to observe objects that were completely hidden behind an opaque scattering layer. However, such approach needs not only to scan both illumination light and detector but further off-line procedures to numerically retrieve the image of the objects. Here, we report a distant invisibility-based noninvasive method that can hide scattering layers and allows to directly image objects behind. By recording holograms of the objects through a ground glass and then using the holograms produced time-reversal lights to re-illuminate the objects, we implemented to observe objects with feature size ranging from 39 μm to 80 μm that were hidden behind a 3 mm thick ground glass. Of importance, our approach opens a door towards real-time, high speed biomedical imaging and in-site inspection of integrated devices.

Imaging through turbid media is an essential observation and diagnostic tool in many fields such as astronomical observations, microscopic imaging in tissues and defects testing of microelectronic devices. Great progress has been made by using methods such as ghost imaging^1^,^2^, adaptive optics technology^3^,^4^,^5^ and wavefront shaping^6^,^7^,^8^,^9^,^10^,^11^,^12^,^13^,^14^,^15^,^16^,^17^,^18^,^19^,^20^,^21^,^22^ to obtain images of objects behind the scattering layers. Among these methods, computational imaging algorithm^1^,^2^,^3^,^4^,^5^,^6^,^7^,^8^, real-time holography^9^,^10^,^11^, interferometry^12^,^13^,^14^,^15^, or focusing and scanning by using spatial light modulator^16^,^17^,^18^,^19^,^20^,^21^,^22^ or ultrasonic technology^23^,^24^ were used. However, most present methods, if not all, are generally invasive because they usually require either a detector or a nonlinear material to be placed behind the scattering layers^6^,^7^,^8^,^9^,^10^,^11^,^15^,^16^,^17^,^18^,^19^,^20^,^21^, or need to acquire knowledge of scattering layers individually in advance so as to reconstruct the images of targets from temporal spectrum^12^,^13^,^14^ [Fig. 1a]. Furthermore, these methods for imaging through scattering layers are generally limited in several hundreds of micrometers in spatial resolutions (no more than 200 μm), and are also not able to resolve details smaller than the thickness or depth of the scattering layers^1^,^2^,^3^,^4^,^5^,^6^,^7^,^8^,^9^,^10^,^11^,^12^,^13^,^14^,^15^,^16^,^17^,^20^,^21^,^22^,^23^,^24^. To overcome these shortcomings, a breakthrough noninvasive method was demonstrated recently to observe a fluorescent object that was completely hidden behind an opaque scattering layer^25^. By scanning the angle of illumination light and collecting scattering light intensity of object in front of the scattering layer, one can retrieve the image of the object through an off-line computation procedure [Fig. 1b]. Obviously, such method undergoes shortcomings like time-consuming and relative low speed. Although a great progress has been made in improving speed by using a single-shot imaging method most recently^26^, it still needs an off-line computation procedure. Consequently, this method is not suitable for real-time imaging and in-site testing.

Here, we describe a noninvasive and robust optical approach to directly observe amplitude–only modulated objects with feature size much smaller than 100 μm (39 μm – 80 μm) deeply behind a turbid medium several millimeters (3 mm in our case) based upon the principle of complementary medium-based distant invisibility^27^,^28^,^29^,^30^. We illuminate objects with a laser beam that has passed through a piece of ground glass and record the interference of reflection light of the objects in front of the ground glass with a reference beam. By using a conjugated object light produced by the interference pattern to re-illuminate the objects through the ground glass, we can conceal the scattering effect of the ground glass and make the hidden objects visible [Fig. 1c]. Our approach needs neither any scanning technologies nor off-line processing procedures such as computer composition or iterative operation and can directly image reflection of objects through a scattering layer in front.

## Results

### Theoretical basis

**Figure 2:**
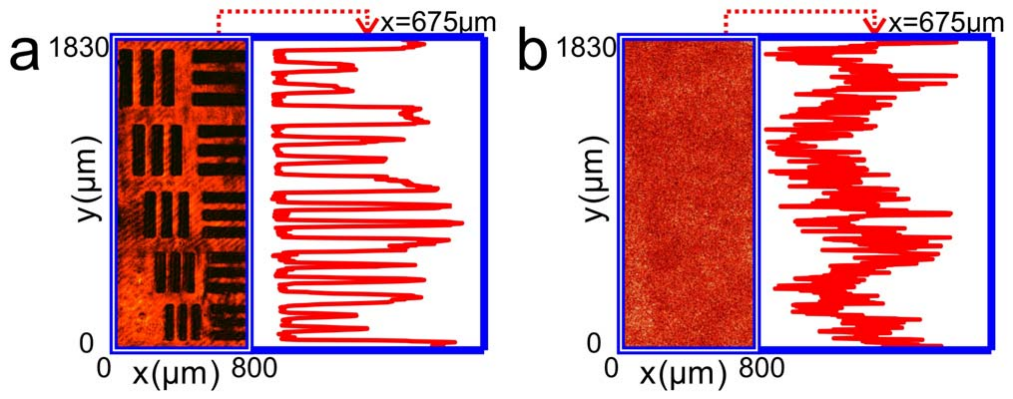
Photographs of a copy of 1951 USAF resolution target used in the experiments. (a), Photograph of the resolution target illuminated by a He-Ne laser beam directly. (b), Photograph of the resolution target illuminated by a He-Ne laser beam through a ground glass. The right hand panels of (a) and (b) are the profiles of intensity distribution of the images along the 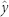 axis at *x* = 675 μm.

From Fourier optics we know that when an object (Fig. 2a) is placed behind a piece of turbid medium (thickness *l*, no absorption is assumed), reflection angular spectrum (AS) of the object through the turbid layer in front can be read as
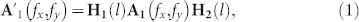
where **H_1_**(*l*) and **H_2_**(*l*) denote the transfer functions (TFs) of turbid medium as illumination light and reflection light of object passing through it, respectively), 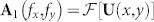 is the AS of object and **U**(*x*, *y*) is the complex amplitude of the object, 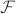 represents the Fourier transform operator, *f_x_* and *f_y_* are the spatial frequencies of the object in the *x* axis and *y* axis, respectively. We see that the information of turbid medium is involved in the reflection signal of object and hence may make the object behind the turbid medium invisible (Fig. 2b).

To clearly image an object through a turbid medium, TFs of the scattering of the turbid medium must be removed from equation (1), i.e. scattering effect of the turbid medium must be concealed and be invisible to the detection devices. In the past few years, several kinds of invisibility cloaks to make a target invisible have been theoretically and experimentally demonstrated^27^,^28^,^29^,^30^,^31^,^32^,^33^. Here we will employ the invisibility principle to realize an opposite function to hide the surroundings and to make the surrounded target visible. By directionally concealing scattering effect of the turbid media to light, we expect to image objects behind the turbid media. Recently, by using multiple light sources placed at different positions to illuminate a phase-modulated surrounding and objects inside in different directions, scientists have realized a function of hiding the surroundings to make the objects inside visible^34^. However, the position of each light source was strictly pre-determined through several times pre-detections. This method in principle is to use phase-conjugation technology to compensate for the scattered light fields of the surroundings. Optically, time-reversal principle was widely exploited to produce phase-conjugation signals for imaging beyond the diffraction limit and for focusing light into a scattering medium^18^,^19^,^21^,^35^, and also for directionally hiding objects and creating optical illusions^29^,^30^. However, previous methods were using phase-conjugation technology to compensate for transmission signals: light sources and detectors/observers were at two sides of the scattering layers or the objects to be hidden, and hence they are an invasive technology. Here, we will use the time-reversal principle to noninvasively image objects through a turbid layer in front.

In methods for optically producing time-reversed light, both linear^10^,^11^ and nonlinear optical effects^9^ can be employed. We employ holography to produce the time-reversed light. We record a hologram of reflection light of the object illuminated by a laser light through the scattering layer with another plane reference light with the complex amplitude **R** in front of the scattering layer. The transmittance of the hologram can be written as 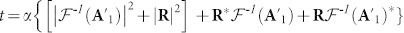, where *α* is a coefficient related to the recording medium and processing procedures of the hologram, superscript * denotes the conjugation operation. When a conjugated reference light R* is used to illuminate the hologram, we can get a transmission light with complex amplitude 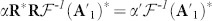 (*α*′ is a real constant). From this formula we can find that the inverse Fourier transform of above transmission light holds an angular spectrum
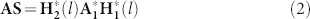
By using this light to illuminate object through the scattering layer, we get reflection AS of object in front of the scattering layer with
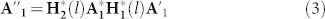
From equation (3) we see that, if both the object and scattering layer are all phase-only modulated, we will not able to observe the object and scattering layer simultaneously. In our case, we assumed that the objects to be imaged are amplitude-only modulated while the scattering layer is absorption-free (phase-only modulated). Hence the complex amplitude **U**(*x*, *y*) of object is a real number and correspondingly, AS of the object is Hermitian 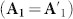
36. Substituting equation (1) into equation (3) and using 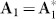, we get reflection AS of the object 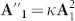, where *κ* is a real coefficient. We see that the reflection signal of the object no longer includes scattering information of the turbid medium. As a result, by inverse Fourier transform (denoted by 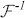) to 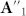, we will obtain a clear image of the object behind the scattering layer. Note that, although mathematically, 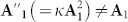, the assumption of amplitude-only modulation of the object indicates that complex amplitude of the object can be regarded as a two-valued function with either **U** = 1 or **U** = 0. Therefore, after normalization we can derive out 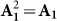
36. Our computer simulations confirm that, if **A_1_** is Hermitian, then 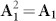. Consequently, by inverse Fourier transform, we get 
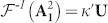
 (*κ*′ is a real coefficient).

### Experimental scheme

To verify the conclusion above, we set up an optical system (Fig. S1) to record on-axis (off-axis, dashed lines) holograms of the objects reflected through a turbid layer in front. The laser beam is from a He-Ne laser (632.8 nm, 70-mW). The object we used at first is a copy of 1951 USAF resolution target (with stripes widths and spaces ranging from 39 μm to 63 μm) made on a glass substrate (Edmund Optics Inc.). Figure 2a shows image of the copied resolution target taken by a charge coupled device (CCD) when it is directly illuminated by a He-Ne laser beam. The profile of intensity distribution of the image along the 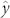 axis at *x* = 675 μm is shown in the right hand panel of the figure, where the notches and peaks correspond to the stripes and spaces between the stripes of the resolution target, respectively. The turbid medium is a piece of 3 mm thick acidized ground glass (For details of the fabrication process, see Supplementary information) with size of scattering particles larger than 200 μm (Fig. S2). Figure 2b shows reflection image of the target through the ground glass as it is illuminated by the laser beam. The right hand panel shows the profile of intensity distribution of the image along the 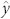 axis at *x* = 675 μm. We see that all the stripes of the target are blurred and the intensity notches and peaks corresponding to the stripes and spaces between the stripes of the resolution target, respectively, are not able to discriminate.

By introducing a reference light to interfere with the reflection light of object through the ground glass in front, we can use a photosensitive medium to record the interference pattern to obtain a hologram. The completed hologram (For details of the fabrication process, see Supplementary information) is re-placed at its original position of recording process and is illuminated by the conjugated reference light. A CCD in front of the ground glass is used to monitor and receive image of the object. Note that both the illumination light source and CCD are in front of the ground glass and hence our method is noninvasive.

### Performance of imaging objects through a turbid medium

**Figure 3:**
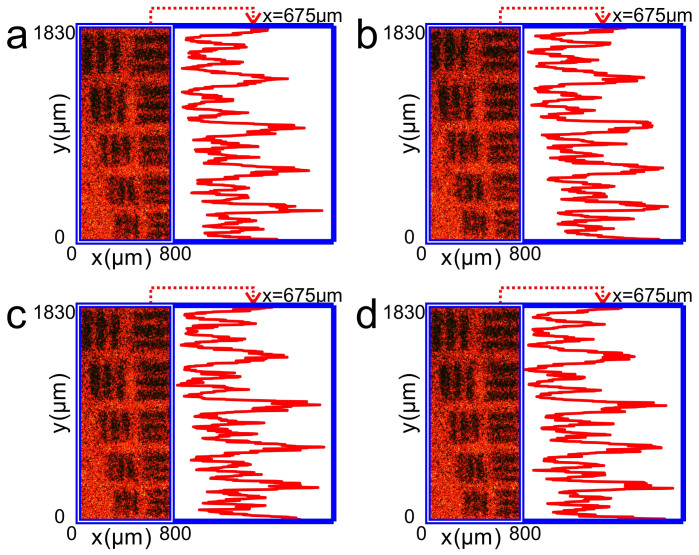
Experimental results of imaging a copy of 1951 USAF resolution target behind a ground glass by using the on-axis holography. (a), Photograph of the resolution target behind a ground glass when a conjugated reference light transmits a hologram to illuminate the resolution target through the ground glass. (b), Photograph of the resolution target behind a ground glass when the hologram is deviated transversally −5 μm away from the original position along the 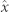 axis. (c) and (d) Photographs of the resolution target behind a ground glass when the hologram is deviated longitudinally ±50 μm (approaching to or going away from the object), respectively, away from the original plane along the 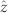 axis. The right hand panels of (a)–(d) are the intensity distributions of the images along the 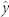 axis at *x* = 675 μm.

Figure 3a shows reflection image of the resolution target through the ground glass when illumination light is a conjugated light of the reference beam after passing through the hologram in the on-axis case. We can see that the object is re-visible again. The profile of intensity distribution of the image along the 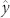 axis at *x* = 675 μm (right hand panel) shows that the intensity notches and peaks corresponding to the stripes and spaces between the stripes of the resolution target, respectively, are clearly discriminated.

In holographic conjugation technology, deviations of the re-placed hologram from its original position and orientation etc. in the recording process may seriously destroy the function of the technology. We then check the effect of position and orientation deviations on the image quality in our approach. Figure 3b shows image of the object through the ground glass as the hologram deviates transversally −5 μm away from its original position along the 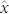 axis. The right hand panel is the intensity profile of the image along the 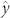 axis at *x* = 675 μm. We can see that all the stripes are still clearly visible. When the transversal deviation is larger than −5 μm away from its original position in the 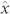 axis, the thinnest stripes (39 μm) begin to become blurring. As the transversal deviation becomes as large as −100 μm away from its original position, the thickest stripes (63 μm) also turn to be not readable. A movie shows the dynamic revolution of the thinnest (39 μm) and thickest (63 μm) stripes of the resolution target from clear to blur (Movie S1).

Figures 3c and 3d show the reflection images of objects through the ground glass and the corresponding intensity profiles (right hand panels) as the hologram deviates longitudinally ±50 μm (approaching to or going away from the object) away from its original plane along the 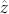 axis, respectively. We find that such large deviation is still tolerable and the thinnest stripes are discriminated. If the deviation becomes larger than ±50 μm, similar to that occur in transversal deviation, the stripes from the thinnest (39 μm) to the thickest (63 μm) will gradually turn into blur as the deviation is as large as ±1.7 mm away from its original position (Movies S2 and S3).

For the effect of orientation deviation of hologram on image quality, we find that a little deviation from its original orientation will seriously destroy the images of the resolution target. Our experimental results show that when orientation deviation of the hologram is as small as 0.1° away from its original orientation around the 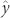 axis in the 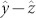 plane (the smallest step of our step motor), image of the resolution target through the ground glass will not be readable completely.

**Figure 4:**
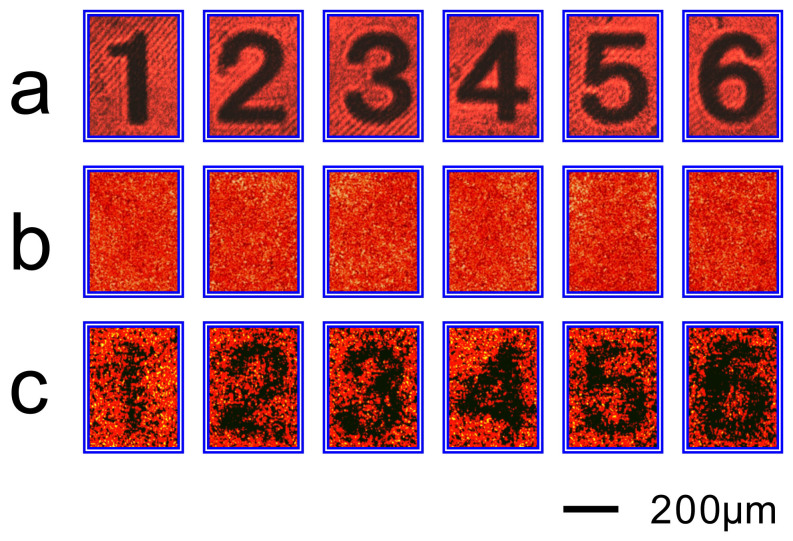
Experiment results of imaging the numbers behind a ground glass by using the on-axis holography. (a), Photographs of the numbers to be imaged behind a ground glass. (b), Photographs of the numbers behind a ground glass when they are illuminated directly by a He-Ne laser beam. (c), Photographs of the numbers when they are illuminated through the ground glass by a conjugated light produced by the corresponding hologram of each number.

Further, we check the reliability of our method in imaging objects with a little more complicated structure, such as the numbers “1, 2, 3, 4, 5, 6” with 80 μm linewidth (Fig. 4a). The holograms of each number were fabricated with the same optical setup and processing procedures as that of resolution target. Figure 4b shows the images of the numbers through the ground glass, where we cannot read any information of the numbers. However, when a conjugated reference light after passing through the hologram of a number is used to illuminate the number through the ground glass, we can obtain a much clearer image of the number behind the turbid glass (Fig. 4c).

**Figure 5:**
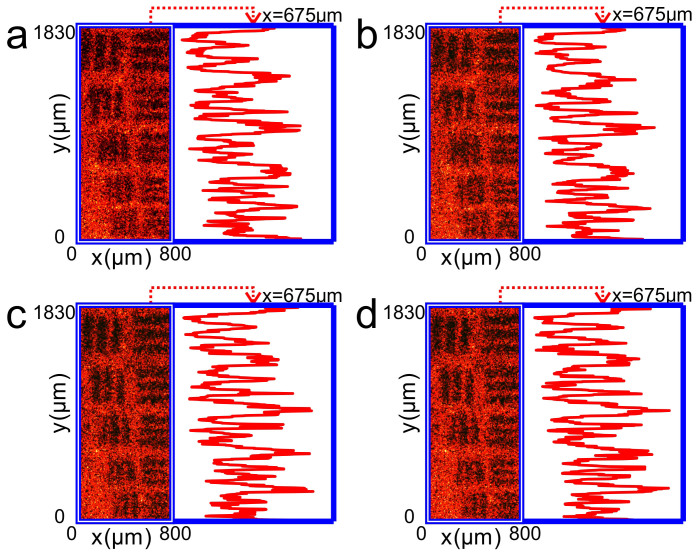
Experimental results of imaging a copy of 1951 USAF resolution target behind a ground glass by using the off-axis holography. (a), Photograph of the resolution target behind a ground glass when a conjugated reference light transmits a hologram to illuminate the resolution target through the ground glass. (b), Photograph of the resolution target behind a ground glass when the hologram is deviated transversally −5 μm away from the original position along the 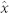. (c), and (d), Photographs of the resolution target behind a ground glass when the hologram is deviated longitudinally ±50 μm (approaching to or going away from the object), respectively, away from the original plane along the 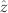 axis. The right hand panels of (a)–(d) are the intensity distributions of the images along the 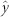 axis at *x* = 675 μm.

**Figure 6:**
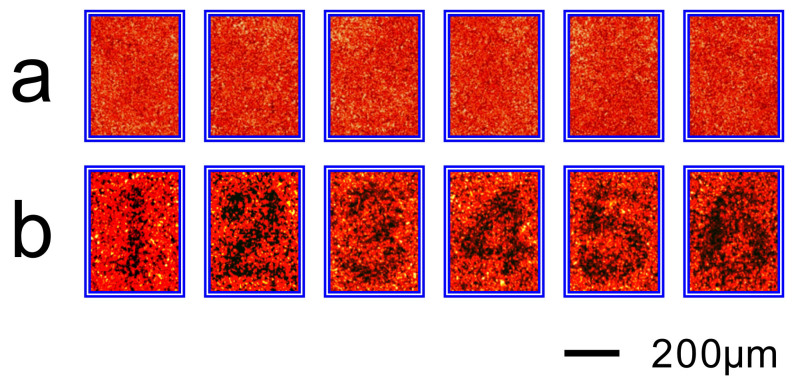
Experiment results of imaging the numbers behind a ground glass by using the off-axis holography. (a), Photographs of the numbers behind a ground glass when they are illuminated directly by a He-Ne laser beam. (b), Photographs of the numbers when they are illuminated through the ground glass by a conjugated light produced by the corresponding hologram of each number.

Figures 5 and 6 exhibit the same as in Figs. 3 and 4, respectively, but are obtained by an off-axis optical system (dashed lines, Fig. S1). Movies S4, S5 and S6 show the dynamic revolution of the images of resolution target from clear to blur. The experimental results show that the off-axis optical system also works well for imaging through the turbid medium.

All experimental results (Figs. 3–6) agree well with the numerical simulations based on computer generated holography (Figs. S4–S7), indicating that both numerical and experimental results verify that our approach to noninvasively image through strong scattering media is feasible.

## Discussions

It should be pointed out that the present method is based on the assumption that the objected we want to observe is amplitude-only modulated while the scattering layer has to produce a phase-only modulation. Hence our method is limited to focus on some special fields such as the observations of cancer cells in biological tissues and bright stars through atmospheric turbulence, where the ill cells and stars are generally treated as absorbing objects or luminous objects with amplitude-only modulation^1^,^2^,^3^,^4^,^5^,^6^,^7^,^8^,^9^,^10^,^11^,^12^,^13^,^14^,^15^,^16^,^17^,^18^,^19^,^20^,^21^,^22^,^23^,^24^, while biological tissues^5^,^8^,^9^,^10^,^11^,^17^ and fogs and clouds^4^,^5^,^17^ are always regarded as non-absorbing media with inhomogeneous refractive-index distributions, which just scatter light and lead to distortion of wave fronts but without energy lost.

On the other hand, the scattering layer should remain unchanged when the hologram is calculated or fabricated as well as the measurement is implemented, which is a quite strong requirement for the technique aiming to noninvasive, and hence to in vivo imaging. To do so, one has to create a conjugation light immediately for imaging the objects behind a scattering layer, which greatly challenges the current materials and technologies. For example, Fe-doped LiNbO3 crystal, one of the typical photorefractive materials for widely using in real-time holography, shows a response time of about 2 min, and has been used to produce optical phase conjugation for turbidity suppression in biological samples^9^. Although spatial light modulators generally hold much shorter response time (about 100 ms) than photorefractive crystals, they need to consume a lot of time in calculation process. Even by using an optimized algorithm, they also need a duration of about 10 s to complete the imaging procedure^17^. The state of the art methods for imaging dynamic/moving object inside/through scattering layer still assume the scattering layer to be unchanged in a certain of time duration^21^,^22^, where the response time is around 2.6 s^21^. Therefore, with the help of real-time holography, our method should be suitable for systems with a little slow variation process such as imaging through biological tissues, monitoring evolution process of organs, as well as defects testing of microelectronic and optical devices etc.

To conclude, we described both theoretically and experimentally a noninvasive imaging method to directly observe amplitude-only modulated objects behind a turbid medium by using holography-based time-reversal principle. Compared to the recent breakthrough made in noninvasive imaging approach to observe a fluorescent object that was completely hidden behind an opaque scattering layer^25^,^26^, our method needs neither multiple direction illuminations and detections nor any off-line computer-aided calculations. We hence believe that our method may be of great interest to generalists and specialists in many disciplines ranging from the life sciences and nanotechnology for real-time, high speed biomedical imaging of biological tissues and in-site inspection of integrated devices.

## Methods

### Recording materials and postprocessing procedures for holograms

The laser is a 70-mW He–Ne laser irradiated at 632.8 nm. Exposure in the recording process of holograms is about 35 μJ/cm^2^. Recording medium is a silver halide holographic recording plate (Tianjin GS-I Holographic Recording Plate, Yiderunfeng Merchandise Trade Ltd., Tianjin, China). To record the holograms, we put the holographic recording plates in the “hologram” plane for exposure (Fig. S1). Formulas of developer, fixer, and bleaching solution for post-processing are shown in Table S1 and the detailed processing schedule is shown in Table S2.

### Fabrications of turbid medium and performance evaluation of holograms

The turbid medium used in the experiment is a piece of 3 mm thick acidized ground glass. The acidized ground glass is prepared by etching one surface of a slide glass in hydrogen fluride liquid (mass concentration 40%, 50 ml) for 25 minutes at 20°C. Figure S2 shows the optical microscope photograph of the acidized ground glass.

To evaluate the performance of holograms for hiding scattering in imaging objects through a ground glass, a step motor with 2.5 μm/step is used to control the movement and orientation of the holograms. A CCD camera is used to record the image and monitor the dynamics of the objects through the ground glass in front (Movies S1~S6).

### Simulation steps for the recording and reconstruction of holograms

1. Choosing the object (a part of 1951 USAF resolution target and the numbers “1, 2, 3, 4, 5, and 6” with 512 × 512 pixels are assumed to be amplitude-only modulated in our case).
2. Calculating the object wavefront after a turbid medium (described as a random phase matrix of 512 × 512) by Fast Fourier Transform (FFT) algorithm.
3. Encoding the calculated wavefront and reference light into a computer generated hologram (with 512 × 512 pixels) by Fresnel diffraction algorithm (containing a FFT).
4. Optical decoding of the transmittance of hologram by using a reconstruction light (conjugated to the reference light) through Fresnel diffraction.
5. Optical reconstruction of the image behind turbid medium by inverse FFT algorithm. The simulated results are shown in Figs. S3–S7.

## Author Contributions

K.D.W. performed the theoretical analysis and experiments, Q.L.C. performed the experiments and simulations, Y.L.S. assisted the experiments, H.W. and G.P.W. designed and conducted the experiments, G.P.W. conceived the idea and supervised the project. All authors contributed to the final version of the manuscript.

## Supplementary Material

Supplementary Information Supplementary Video S1

Supplementary Information Supplementary Video S2

Supplementary Information Supplementary Video S3

Supplementary Information Supplementary Video S4

Supplementary Information Supplementary Video S5

Supplementary Information Supplementary Video S6

Supplementary Information SUPPLEMENTARY INFO

## Figures and Tables

**Figure 1:**
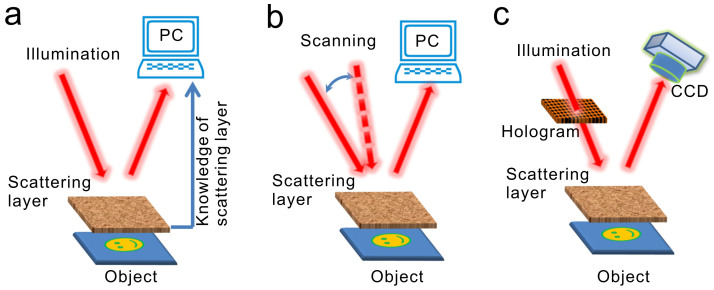
Schematics of experiments for imaging through a scattering layer. (a), Conventional imaging methods, where knowledge of scattering layer is generally needed to acquire individually for further reconstruction of images of objects. (b), A noninvasive imaging method through scanning illumination light and detector as well as image retrieving of an off-line computation procedure. (c), Present noninvasive imaging method. Time-reversal light generated by a hologram is used to hide scattering layer for directly imaging objects hidden by the scattering layer.
