# Supplementary material for: Hiding scattering layers for noninvasive imaging of hidden objects
Source: Sci Rep. 2015 Feb 11;5:8375. doi: 10.1038/srep08375 (PMC4323654; doi:10.1038/srep08375)
Supplement: Supplementary Information — SUPPLEMENTARY INFO [file srep08375-s7.pdf]

SUPPLEMENTARY INFORMATION

Hiding scattering layers for noninvasive imaging of hidden objects

Authors: Kedi Wu[1,2†], Qiluan Cheng[2†], Yile Shi[3], Hui Wang[3], and Guo Ping Wang[1,2*]

Affiliations:

[1] College of Electronic Science and Technology, Shenzhen University, Shenzhen 518060, China

[2] School of Physics and Technology, Wuhan University, Wuhan 430072, China

[3] Institute of Information Optics, Zhejiang Normal University, Jinhua 321000, Zhejiang, P. R. China

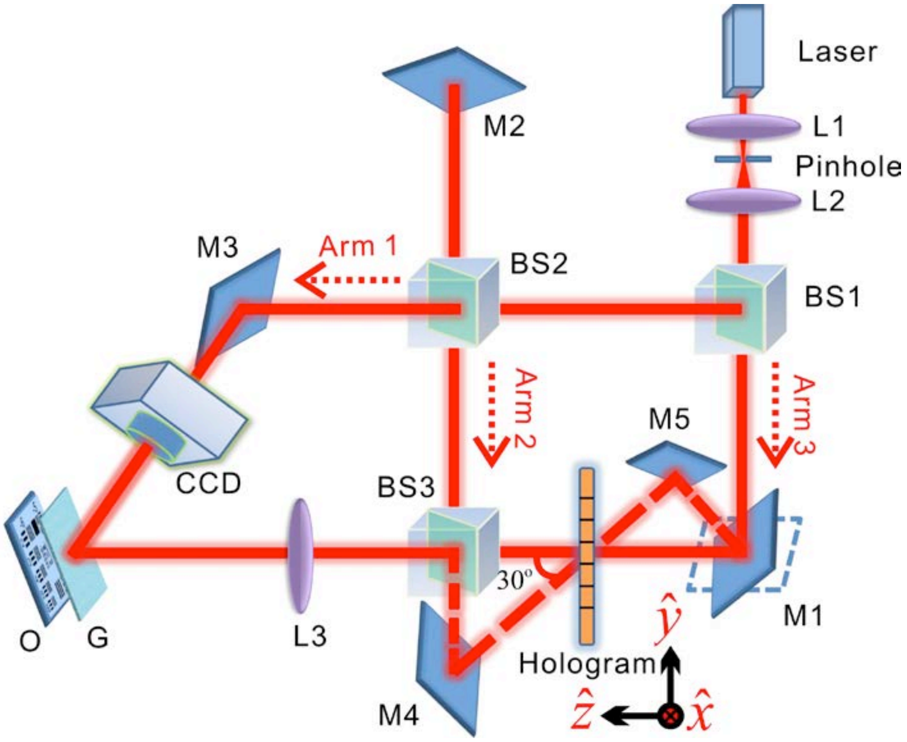

Figure. S1. Experimental setup for imaging object O behind a ground glass G by on-axis (solid lines) and off-axis (dashed lines) holography. A He–Ne laser beam is expanded by lenses L1 and L2, and then divided into three parts by beam splitters BS1 and BS2. Light along the Arm1 is reflected by mirror M3 to illuminate object (1951 USAF resolution target or the numbers in our cases) through the ground glass. Scattering light passes through L3 and then interferes with reference light (reflected light of Arm 2 by BS3 in

the on-axis case, or by M4 in the off-axis case to produce a 30° angle between the scattering light, dashed lines). A holographic recording plate placed in the "hologram" plane is used to record the interference pattern. Reflection lights of Arm 3 by M1 (on-axis) or M1 and M5 (off-axis, dashed lines) are the conjugated beam to the reference lights, which are used to illuminate objects through the ground glass in the observation process. A CCD camera is used to capture the image of objects behind the ground glass.

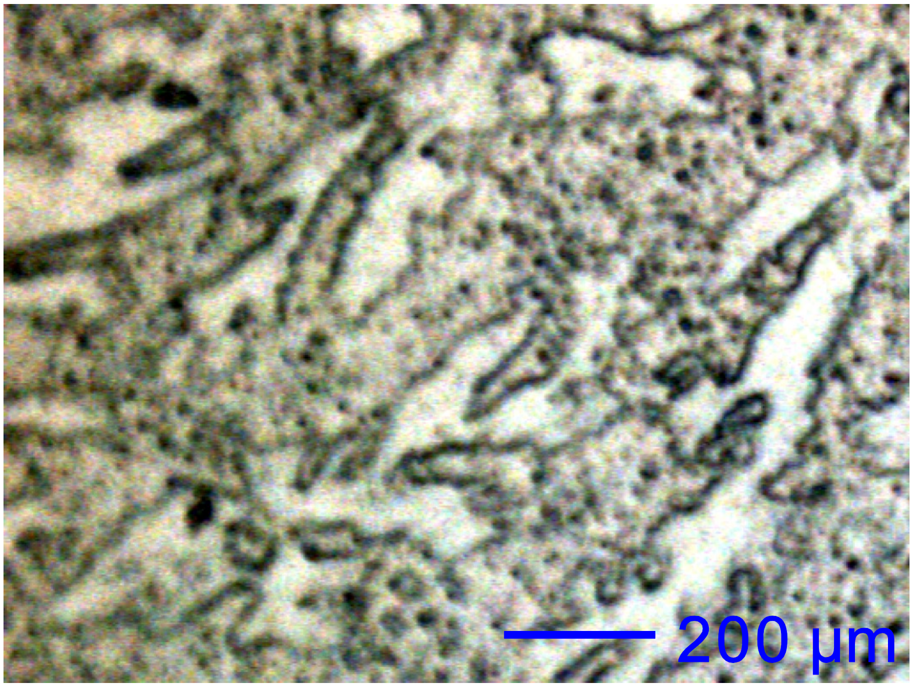

Figure. S2. Photograph of the surface of a acidized ground glass used in the experiments.

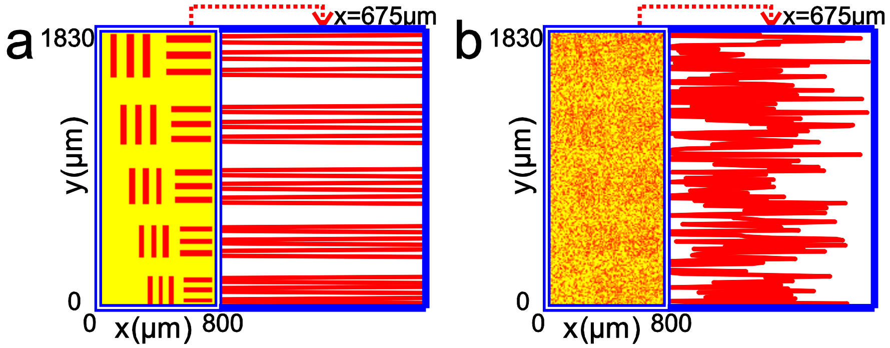

Figure. S3. Simulated results of object behind a turbid medium. **a.** Image of a 1951 USAF resolution target illuminated by a 632.8 nm plane light. **b.** Image of the resolution target behind a ground glass. The right hand panels of (**a**) and (**b**) are the profiles of intensity distribution of the images along the $\hat{y}$ axis at x=675 μm.

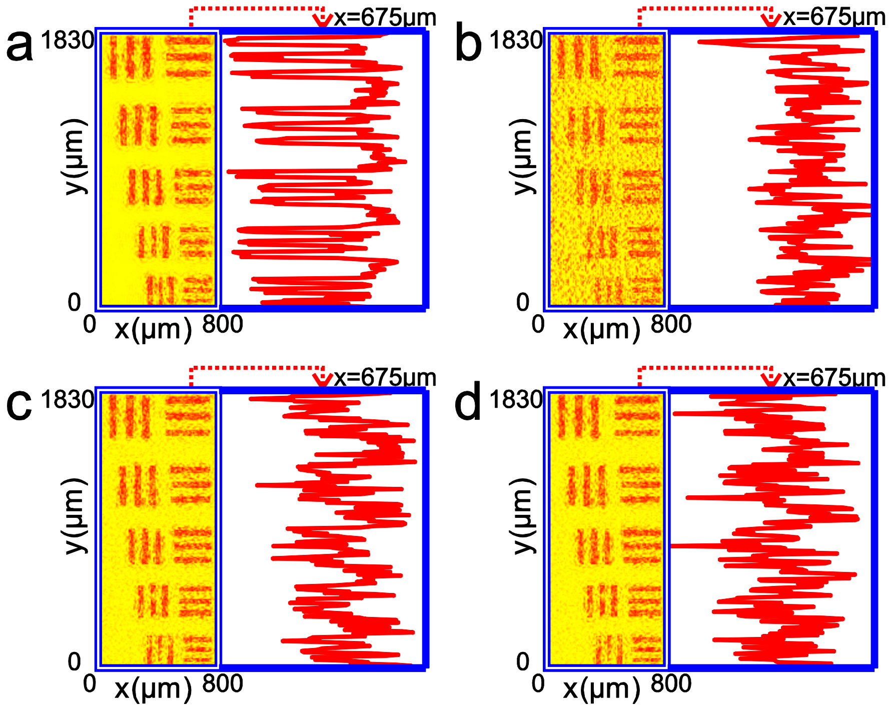

Figure. S4. Simulated results of imaging a 1951 USAF resolution target behind a ground glass by using the on-axis holography. **a.** Image of the resolution target behind a ground glass when a conjugated reference light transmits a hologram to illuminate the resolution target through the ground glass. **b.** Image of the resolution target behind a ground glass when the hologram deviates transversally -5 μm away from the original position along the $\hat{x}$ axis. **c.** and **d.** Images of the resolution target behind a ground glass when the hologram longitudinally deviates ±50 μm (approaching to or going away from the object), respectively, away from the original plane along the $\hat{z}$ axis. The right hand panels of (**a**)-(**d**) are the intensity distributions of the images along the $\hat{y}$ axis at x=675 μm.

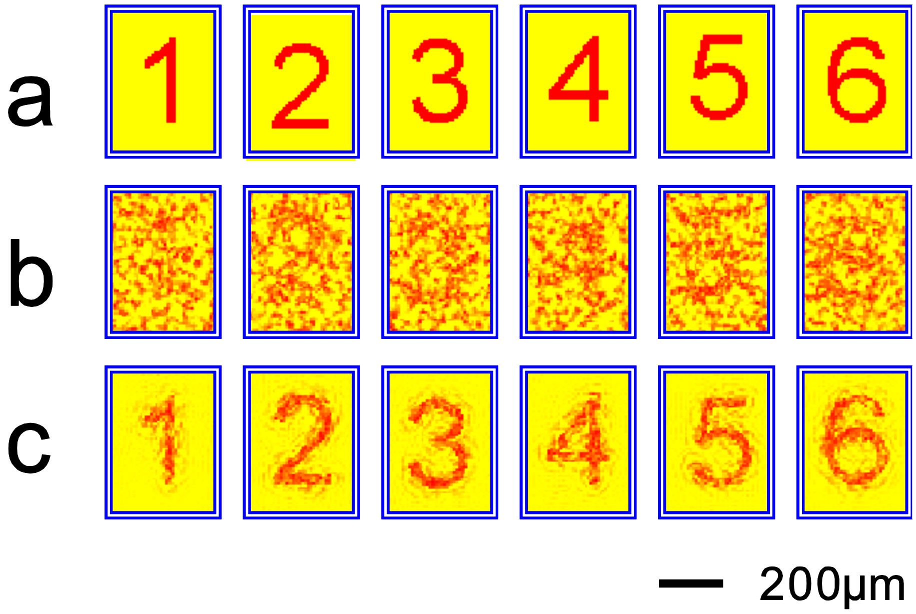

Figure. S5. Simulated results of imaging the numbers behind a ground glass by using the on-axis holography. **a.** The numbers to be imaged behind a ground glass. **b.** Images of the numbers behind a ground glass when they are illuminated directly by a He-Ne laser beam. **c.** Images of the numbers when they are illuminated through the ground glass by a conjugated light produced by the corresponding hologram of each number.

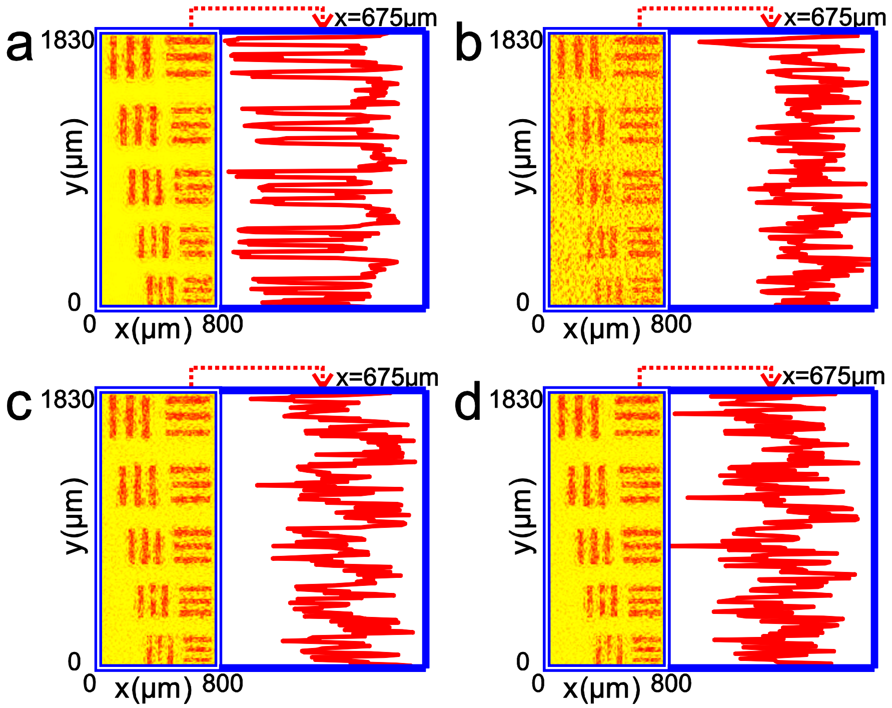

Figure. S6. Simulated results of imaging a1951 USAF resolution target behind a ground glass by using the off-axis holography. **a.** Image of the resolution target behind a ground glass when a conjugated reference light transmits a hologram to illuminate the resolution target through the ground glass. **b.** Image of the resolution target behind a ground glass when the hologram deviates transversally -5 μm away from the original position along the $\hat{x}$ axis. **c.** and **d.** Images of the resolution target behind a ground glass when the hologram longitudinally deviates ±50 μm (approaching to or going away from the object), respectively, away from the original plane along the $\hat{z}$ axis. The right hand panels of (**a**)-(**d**) are the intensity distributions of the images along the $\hat{y}$ axis at x=675 μm.

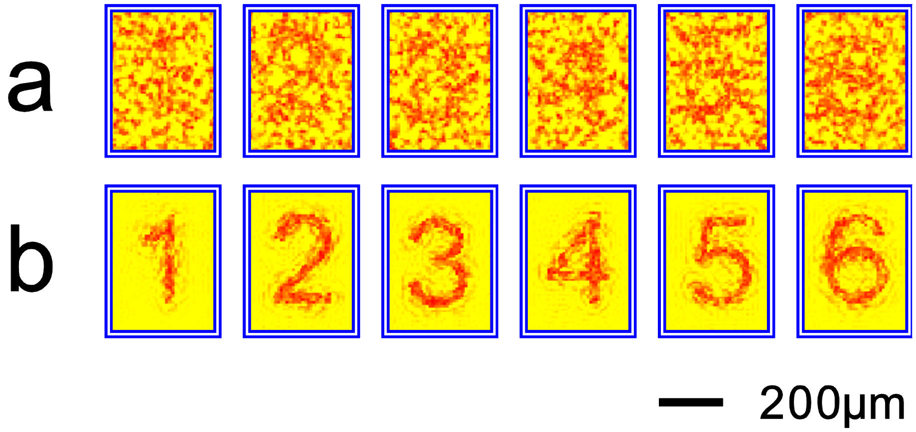

Figure. S7. Simulated results of imaging the numbers behind a ground glass by using the off-axis holography. **a.** Images of the numbers behind a ground glass when they are illuminated directly by a He-Ne laser beam. **b.** Images of the numbers when they are illuminated through the ground glass by a conjugated light produced by the corresponding hologram of each number.

<table>
<tr><td rowspan="2">D-19</td><td>Distilled water</td><td>Metol</td><td>Anhydrous sodium sulfite</td><td>Hydroquinone</td><td>Anhydrous sodium carbonate</td><td>Potassium bromide</td><td>Add distilled water to</td></tr>
<tr><td>800 ml</td><td>2 g</td><td>90 g</td><td>8 g</td><td>48 g</td><td>5 g</td><td>1000 ml</td></tr>
<tr><td rowspan="2">Mercuric chloride bleaching liquid</td><td colspan="2">Distilled water</td><td colspan="2">Mercuric chloride</td><td colspan="2">Potassium bromide</td><td>Add distilled water to</td></tr>
<tr><td colspan="2">500 ml</td><td colspan="2">9 g</td><td colspan="2">4 g</td><td>1000 ml</td></tr>
<tr><td rowspan="2">F-5</td><td>Distilled water (50°C)</td><td>Sodium thiosulfate</td><td>Anhydrous sodium sulfite</td><td>Acetic acid</td><td>Boric acid</td><td>Potassium alum</td><td>Add distilled water to</td></tr>
<tr><td>800 ml</td><td>240 g</td><td>15 g</td><td>13.5 ml</td><td>7.5 g</td><td>15 g</td><td>1000 ml</td></tr>
<tr><td rowspan="4">R-10</td><td rowspan="2">Solution A</td><td>Distilled water</td><td>Ammonium dichromate</td><td>Concentrated sulfuric acid</td><td>Add distilled water to</td><td colspan="2" rowspan="4">Mix one part A and one part B before using.</td></tr>
<tr><td>500 ml</td><td>20 g</td><td>14 ml</td><td>1000 ml</td></tr>
<tr><td rowspan="2">Solution B</td><td colspan="2">Sodium chloride</td><td colspan="2">Add distilled water to</td></tr>
<tr><td colspan="2">45 g</td><td colspan="2">1000 ml</td></tr>
</table>

Table S1: Formulas for D-19 (developer), F-5 (fixer), mercuric chloride bleaching liquid, and R-10 (bleaching solution). All the chemicals in the table were required to be analytically pure.

Operation	Time and temperature
Developed in D-19 (in the darkroom)	<3 min ($20^{\circ}C$)
Rinsed in running water (in the darkroom)	2 min ($18^{\circ}C$-$20^{\circ}C$)
Bleached in mercuric chloride bleaching liquid (in the darkroom)	1 min ($18^{\circ}C$-$20^{\circ}C$)
Rinsed in running water (in the darkroom)	1 min ($18^{\circ}C$-$20^{\circ}C$)
Illuminated by mercury vapor lamp (in the darkroom)	<2 min ($18^{\circ}C$-$20^{\circ}C$)
Fixed in F-5 (in the darkroom)	3 min ($19^{\circ}C$-$20^{\circ}C$)
Rinsed in running water	2 min ($18^{\circ}C$-$20^{\circ}C$)
Bleached in R-10 (1 part of R-10 is diluted by 5 parts of distilled water)	1–2 min ($19^{\circ}C$-$20^{\circ}C$)
Rinse in running water	10 min ($18^{\circ}C$-$20^{\circ}C$)

Table S2: Post-processing procedures. After all the steps listed in this table are completed, the silver halide holographic recording plates are air dried before inserting in the optical path.

Video legends:

Supplementary Videos S1

A video illustrates the dynamic process of imaging part of a copy of 1951 USAF resolution target behind a ground glass by using the on-axis optical path (Fig. S1). Hologram is re-placed with a displacement (S**1**) 0~-100 μm in the hologram plane along the $\hat{x}$ axis (QuickTime; 53 KB).

Supplementary Videos S2

A video illustrates the dynamic process of imaging part of a copy of 1951 USAF resolution target behind a ground glass by using the on-axis optical path (Fig. S1). Hologram is re-placed with a displacement 0~-1.7mm away from the hologram plane along the $\hat{z}$ axis (QuickTime; 122 KB).

Supplementary Videos S3

A video illustrates the dynamic process of imaging part of a copy of 1951 USAF resolution target behind a ground glass by using the on-axis optical path (Fig. S1). Hologram is re-placed with a displacement 0~1.7mm away from the hologram plane along the $\hat{z}$ axis (QuickTime; 129 KB).

Supplementary Videos S4

A video illustrates the dynamic process of imaging part of a copy of 1951 USAF resolution target behind a ground glass by using the off-axis optical path (dashed lines, Fig. S1). Hologram is re-placed with a displacement (**S4**) 0~-100 μm in the hologram plane along the $\hat{x}$ axis (QuickTime; 98 KB).

Supplementary Videos S5

A video illustrates the dynamic process of imaging part of a copy of 1951 USAF resolution target behind a ground glass by using the off-axis optical path (dashed lines, Fig. S1). Hologram is re-placed with a displacement 0~ -1.7mm away from the hologram plane along the $\hat{z}$ axis (QuickTime; 132 KB).

Supplementary Videos S6

A video illustrates the dynamic process of imaging part of a copy of 1951 USAF resolution target behind a ground glass by using the off-axis optical path (dashed lines, Fig. S1). Hologram is re-placed with a displacement 0~1.7mm away from the hologram plane along the $\hat{z}$ axis (QuickTime; 137 KB).
